# Comparison of the Clinical Manifestation of HPAI H5Nx in Different Poultry Types in the Netherlands, 2014–2022

**DOI:** 10.3390/pathogens13040280

**Published:** 2024-03-26

**Authors:** Wendy J. Wolters, J. C. M. Vernooij, Thomas M. Spliethof, Jeanine Wiegel, Armin R. W. Elbers, Marcel A. H. Spierenburg, J. Arjan Stegeman, Francisca C. Velkers

**Affiliations:** 1 Department of Population Health Sciences, Faculty of Veterinary Medicine, Utrecht University, 3584 CL Utrecht, The Netherlands; w.j.wolters@uu.nl (W.J.W.); j.c.m.vernooij@uu.nl (J.C.M.V.);; 2 Division of Pathology, Department of Biomolecular Health Sciences, Faculty of Veterinary Medicine, Utrecht University, 3584 CS Utrecht, The Netherlands; t.m.spliethof@uu.nl; 3 Royal GD, 7418 EZ Deventer, The Netherlands; j.wiegel@gdanimalhealth.com; 4 Department of Epidemiology, Bioinformatics, Animal Studies and Vaccine Development, Wageningen Bioveterinary Research, 8200 AB Lelystad, The Netherlands; armin.elbers@wur.nl; 5 Netherlands Food and Consumer Product Safety Authority (NVWA), 3511 GG Utrecht, The Netherlands

**Keywords:** highly pathogenic avian influenza, poultry, influenza subtypes, disease outbreaks, mortality, clinical signs, host factors

## Abstract

This study describes clinical manifestations of highly pathogenic avian influenza (HPAI) H5N1, H5N8 and H5N6 outbreaks between 2014 and 2018 and 2020 and 2022 in the Netherlands for different poultry types and age groups. Adult duck (breeder) farms and juvenile chicken (broiler and laying pullet) farms were not diagnosed before 2020. Outbreaks in ducks decreased in 2020–2022 vs. 2014–2018, but increased for meat-type poultry. Neurological, locomotor and reproductive tract signs were often observed in ducks, whereas laying- and meat-type poultry more often showed mucosal membrane and skin signs, including cyanosis and hemorrhagic conjunctiva. Juveniles (chickens and ducks) showed neurological and locomotor signs more often than adults. Diarrhea occurred more often in adult chickens and juvenile ducks. Mortality increased exponentially within four days before notification in chickens and ducks, with a more fluctuating trend in ducks and meat-type poultry than in layers. For ducks, a mortality ratio (MR) > 3, compared to the average mortality of the previous week, was reached less often than in chickens. A lower percentage of laying flocks with MR > 3 was found for 2020–2022 vs. 2014–2018, but without significant differences in clinical signs. This study provides a basis for improvements in mortality- and clinical-sign-based early warning criteria, especially for juvenile chickens and ducks.

## 1. Introduction

Highly pathogenic avian influenza (HPAI) is a global problem affecting domestic poultry, wild birds, mammals and sometimes humans [1,2]. Since 2014, H5 clade 2.3.4.4 outbreaks have occurred frequently in poultry and wild birds in Europe, Africa, the Americas and Asia [1,3,4,5,6,7,8]. The 2021–2022 HPAI epidemic in Europe has been the largest HPAI epidemic so far. This epidemic was especially long-lasting, with outbreaks all through the summer, and was directly followed by the 2022–2023 outbreak season [4,5,6,7,8]. The ongoing outbreaks during the summer months and genetic analyses have indicated that the HPAI virus persisted in residential wild birds [8]. Moreover, not only were wild waterfowl (Anseriformes) such as geese, swans and ducks commonly affected, but the HPAI virus was seen in an increasing number of different wild bird species, including raptors (Falconiformes) and gulls, gannets and terns (Charadriiformes, family Laridae). During the 2022–2023 outbreak season, in France, Belgium, Czechia, Germany, Poland, Sweden, the UK, the Netherlands and Italy HPAI virus was prevalent in sea bird breeding colonies, in which gull species such as black-headed gulls showed high mortality [8,9,10].

Compared to 2020–2021, an increase in the number of outbreaks in almost all types of domestic poultry was seen in Europe in 2021–2022, particularly in the number of affected fattening, foie gras and breeding farms (chickens, domestic ducks and geese) [4]. Also, in the Netherlands, a change in the distribution of affected poultry types was seen from 2021 onwards, including outbreaks in broiler, layer-rearing, breeder-duck and turkey farms that had not been seen previously [4,5,6,7,8]. It is unclear whether this change in distribution is just coincidental or associated with differences in management, virus characteristics or host susceptibility [11]. 

Differences in susceptibility to HPAI infection and disease between wild birds and poultry, but also between domestic poultry species, i.e., gallinaeceous (Galliformes) poultry, such as chickens and turkeys, and domestic ducks (Anseriformes), have been described previously [12,13]. Also, age-related differences have been studied in several poultry species [14,15,16,17,18]. For example, differences in mortality and clinical and pathological findings and age-related differences in susceptibility were found in several experimental studies, e.g., with H5N2, H4N8 and H3N2 in layers [16], HPAI and LPAI H5N2 recombinant HA proteins in chickens, ducks and turkeys [17], LPAI H3N1 in pullets [18] and with HPAI H5N1 in ducks [15]. However, in an experiment with HPAI H5N2 clade 2.3.4.4 virus, obtained from the 2015 outbreaks in the United States of America (USA), age did not affect susceptibility, with similar virus shedding and severe pathology in 5-week-old broilers, 8-week-old broilers and >30-week-old broiler breeders [14].

The HPAI H5 2.3.4.4. clade virus strains H5N8, H5N6 and H5N1 have been detected since 2014, and from 2021 onwards H5N1 (clade 2.3.4.4b) has been predominant across Europe. It is known that clinical manifestation may differ between virus strains and, moreover, even within the same clade [19,20]. Therefore, knowing how HPAI clinically manifests in different poultry species, including different age groups and production types, and how this changes over time is highly relevant. Because HPAI notification relies on passive surveillance, this knowledge may help to recognize HPAI infections and detect these virus infections earlier, and can help promote the use of protocols for early detection. Furthermore, it may increase awareness in farmers and veterinarians of the significance of HPAI infections in the various poultry types. Finally, it may provide a better understanding of the pathophysiology of HPAI and the influence of the host, virus and other contributing factors related to the poultry production system, such as management and biosecurity. 

Contributing to this, in this study we (i) described and analyzed the distribution of the different infected poultry species for the different outbreak seasons; (ii) systematically assessed and described the clinical representation and changes in performance parameters in layer-type poultry, meat-type poultry and ducks, using cases of all outbreak seasons together, and studied potential age-dependent influences on the susceptibility for and the clinical manifestation of HPAI; and (iii) evaluated whether the smaller number of cases between 2014 and 2018 versus 2020 and 2022 was associated with changes in virus pathogenicity, for which we focused on the most elaborate dataset of domestic layers. 

## 2. Materials and Methods

### 2.1. Study Population

This study includes data from 92 domestic commercial poultry farms diagnosed with HPAI H5N8, H5N6 and H5N1 in the Netherlands in the seasons 2014–2015, 2016–2017, 2017–2018, 2020–2021 and 2021–2022, which included 4 layer-rearing farms, 35 layer farms, 1 layer breeder farm, 17 broiler farms, 7 broiler breeder farms, 1 grandparent broiler breeder farm, 16 meat duck farms, 5 duck breeder farms and 6 turkey farms (Table 1). Some of these farms (*n* = 11) were infected with HPAI multiple times between the different seasons and were included in the dataset each time they were diagnosed. In line with autumn migration, we assumed that each new bird flu season started on the first of October. The farms are referred to using a farm ID starting with an abbreviation referring to the poultry type, consisting of ‘B’ for broilers, ‘L’ for layers, ‘T’ for turkeys and ‘D’ for Pekin ducks, followed by a consecutive number according to the time of diagnosis. All poultry from HPAI-infected poultry farms were culled within days of the diagnosis. Data from noninfected domestic poultry farms were not included in this study.

### 2.2. Data Collection

HPAI-suspected farms were visited by a veterinary specialist team from the Dutch Competent Authority, the Netherlands Food and Consumer Product Safety Authority (NVWA) and Royal GD (GD, Deventer, The Netherlands) for clinical inspection and an official conformational sampling of poultry in the affected poultry house(s). Most farms became HPAI-suspected after the notification of a clinical suspicion by the farmer or veterinarian to NVWA. Some farms were detected after a positive oropharyngeal or cloacal swab was tested within the Dutch national ‘early warning’ diagnostic framework to rule out LPAI or HPAI (as described previously by Schreuder et al., 2021) [21,22], or after contact tracing or preventive screening when located near other infected farms. Reporting criteria for notification by farmers and veterinarians include thresholds for increased mortality, reduced egg production or feed or water consumption, depending on the poultry type, and are described in Appendix A. 

In affected poultry houses of suspected farms, oropharyngeal and cloacal swabs were taken from 20 birds (preferably from clinically affected birds or recently deceased birds) and tested by the national reference laboratory WBVR (Wageningen Bioveterinary Research, Lelystad, The Netherlands) using matrix-gene real-time PCR [23], followed by subtyping with H5-specific real-time PCR [24] and Sanger sequencing of the HA cleavage site and N-subtype [23]. A standardized form, including the date of onset of clinical signs and a checkbox list of clinical signs, was filled out by the NVWA and the Royal GD veterinarians in cooperation with the poultry farmer (Table S1). On farms with confirmed infections, the NVWA performed a standard epidemiological investigation to identify possible dangerous contacts prior to culling and to collect further information on the clinical history and changes in performance parameters by interviewing the farmer and farmers’ contacts. In addition, 36 out of 92 infected poultry farms were visited for a more detailed epidemiological investigation after culling by specialized poultry veterinarians from the Faculty of Veterinary Medicine of Utrecht University, sometimes together with a veterinarian working for the NVWA or an epidemiologist from WBVR (Table 2). This included the filling out of a previously developed questionnaire [21] by the researchers during a detailed interview with the farmer, in combination with an on-site evaluation of the farm premises and the surrounding area, to discover potential routes for virus introduction and weaknesses in biosecurity. Most farms (25 of 26 farms) were visited before the 2021–2022 season, but due to the large number of infected farms only 11 out of 66 farms in the 2021–2022 season were visited (Table 2). For the other farms (*n* = 55), reports of farm visits from the NVWA, Royal GD and epidemiologists of WBVR were used if available, and supplemented with interviews with farm veterinarians, Royal GD veterinarians and other farm visitors and data collected by a voluntary online survey of farmers (response rate *n* = 16). 

### 2.3. Clinical Signs

The standardized form, with the date of onset of clinical signs and the checkbox list of clinical signs during the official inspection and sampling visit, was retrieved from the NVWA for 83 out of 92 farms and the Royal GD for 61 out of 92 farms (Table 2 and Table S1). Additional and more detailed information on the clinical history obtained from the farmers during farm visits, farm visit reports and interviews was used to supplement the dataset. The clinical signs were categorized into six groups of clinical signs as described previously [21], i.e., general signs, and signs from the respiratory system, nervous and locomotor system, mucosal membranes and skin, gastrointestinal tract or reproductive tract. General signs included reduced feed and/or water intake, decreased activity or lethargy, hunched posture, ruffled feathers and sudden death. The signs included in the other categories are summarized in Table S2. 

### 2.4. Mortality Ratio and Performance Parameters

Farm production charts collected by the NVWA during the epidemiological investigation, containing at least the daily records of mortality, and in some cases also feed and water intake and egg production for egg-producing poultry, were retrieved for 63 out of 92 farms (Table 2). At seven farms, multiple houses were infected. The percentage of daily mortality was calculated per house. To avoid calculation problems when taking log10 of the mortality percentage, 0.1 was added to all percentages, resulting in the lowest value of log10(0.1) = −1 indicating an absence of mortality (0%). Mortality ratios (MRs) of the day before notification were calculated for each flock using the method described by Gonzales and Elbers (2018) [25]. This method was also applied by Schreuder et al. (2021) [21] on a similar dataset, but for this study we slightly adjusted the method, using a threshold >3 instead of >2.9. This threshold was chosen to be in line with current Dutch legislation (see Appendix A) that indicates a need to notify the competent authority when an increase in daily mortality >3 times the average mortality of the preceding week is present for two consecutive days in layers, breeders and broilers ≥10 days. 

For each farm, all available NVWA and Royal GD reports and data from interviews were used to determine whether changes in egg production (production percentage, but also presence of pale or mottled eggs and reduced egg-shell quality) were observed. These changes were categorized as the presence or absence of signs of the reproductive tract. Changes in feed and water intake were categorized under general clinical signs.

### 2.5. Data Analysis 

The clinical and diagnostic data of all infected farms were first visualized using descriptive analyses. All analyses were performed with software package R version 4.2.2 [26]. 

As the number of infected farms for each individual poultry production type was limited, farms were categorized based on genetic background into either laying- or meat-type gallinaceous (fowl-like) poultry from the order Galliformes, and waterfowl from the order Anseriformes (Table 3). The three categories consisted of 1: Galliformes layer type poultry (layer-rearing, layer); 2: Galliformes meat-type poultry (broiler, broiler breeder, turkey); and 3: Anseriformes (meat duck, duck breeder). A further categorization was made to study potential age-related differences in susceptibility to HPAI. The infected poultry farms, except for the small number of turkey farms (*n* = 6), were categorized as 1A + 2A: chicken juvenile (layer rearing and broiler); 1B + 2B: chicken adult (layer, layer-breeder, broiler breeder/grandparent); 3A: duck juvenile (meat duck); and 3B: duck adult (duck breeder). See Table 3 for the categorization and number of farms for each of these categories.

The infected farms were classified into two time periods, i.e., those diagnosed between 2014 and 2018 and between 2020 and 2022. The first period included infections with H5N8 virus in 2014–2015 and 2016–2017, and H5N6 in 2017–2018. In the second period, H5N8 and H5N1 virus were detected in 2020–2021 and 2021–2022, respectively. 

The six categories of clinical signs (i.e., general signs, signs of the nervous or locomotor system, signs of mucosal membranes and skin, respiratory signs, gastrointestinal signs and reproductive signs) and whether a MR > 3 was present on the day prior to notification were included in the dataset as binary variables (present or absent or unknown). 

Fisher’s exact tests were used to study associations between the distribution of infected poultry farms and poultry type (laying-type, meat-type or ducks) and ages (chicken adult, chicken juvenile, duck adult, duck juvenile). Fisher’s exact tests were also applied for the observed presence of each of the six categories of clinical signs and poultry type (laying-type, meat-type or duck), ages (chicken adult, chicken juvenile, duck adult, duck juvenile) and layer farms (infected in 2014–2018 vs. 2020–2022). As only adult poultry can produce eggs, signs of the reproductive tract were not included in the analyses of the clinical signs for the different ages in Section 3.2.2. Fisher’s exact tests were also used to evaluate associations between each of the clinical signs and MR > 3 for the outbreaks of seasons in 2014–2018 compared to 2020–2022. The level of significance was set at 0.05. 

## 3. Results

### 3.1. Type of Infected Domestic Poultry Farms for the Different Outbreak Seasons

The total number of outbreaks on domestic poultry farms varied between the outbreak seasons, with many cases in 2021–2022 in particular (Table 1 and Figure S1). In 2015–2016 and 2018–2019, there were no cases of infected poultry farms. Over the years, three different virus strains have been detected, but from 2021 onwards only H5N1 was diagnosed (Table 1). Most farms were located in the western and northern parts of the Netherlands, near the coast, as well as around the larger rivers in the center of the country (e.g., the Rhine, Waal, Ijssel and Meuse rivers), as described in previous studies [3,21,27,28,29,30,31] and visualized on the website of WBVR [32]. Due to the almost continuous mandatory indoor confinement regulations in the study period, most farms, including laying hen farms with free range systems, had to keep the chickens inside.

Significant differences in the distribution of the types of infected poultry farms were found for 2020–2022 compared to 2014–2018, with more meat-type poultry farms (broiler, broiler breeder, turkey) (39.5% vs. 6.3%) and fewer duck farms (18.4% vs. 43.8%) affected after 2020 (Table 4). Turkey and broiler farms were not diagnosed with HPAI until 2020 (Table 1). Layer-rearing, broiler grandparent and duck breeder farms were not diagnosed until 2021 (Table 1). Also, significant differences in the distribution of the affected age groups of chicken and duck farms were found. Between 2014 and 2018, only juvenile ducks and adult chickens were diagnosed with HPAI, whereas since 2020 juvenile chickens (layer rearing and broiler farms) and adult ducks (duck breeder farms) have also been diagnosed (Table 1 and Table 4). The percentage of meat duck farms was lower (13.0% vs. 43.8%) from 2020 onwards compared to 2014–2018 (Table 4). 

### 3.2. Clinical Manifestation of HPAI

#### 3.2.1. Production Types

No difference was found for signs of general disease and signs attributed to the gastrointestinal tract between meat-type poultry, layer-type poultry and ducks (Table 5). However, reduced feed intake, which was categorized as one of the signs of general disease, was mainly seen in ducks (Figures S2–S4). Signs of the nervous and locomotor system were often seen on duck farms (67.7%), but were also reported for meat-type poultry (48.4%), and less frequently in layer-type poultry (15.0%) (Table 5, *p* < 0.001, Figures S2–S4). In contrast, signs attributed to the mucosal membranes and skin were mainly seen in layer- (55.0%) and meat-type poultry (67.8%) and on only 4.7% of duck farms (Table 5, *p* < 0.001, Figures S2–S4). Respiratory signs were most often seen in meat-type poultry (61.3%) compared to layers (37.5%) and ducks (23.8%) (Table 5, *p* = 0.02, Figures S2–S4). Signs of the reproductive tract were mainly seen in ducks (100%) but less in layer-type poultry (19.4%) and meat-type poultry (28.6%) (Table 5, *p* < 0.001, Figures S2–S4), as the latter category also contained many broiler and turkey farms that do not produce eggs (Table 3). 

#### 3.2.2. Age

**Figure 1:**
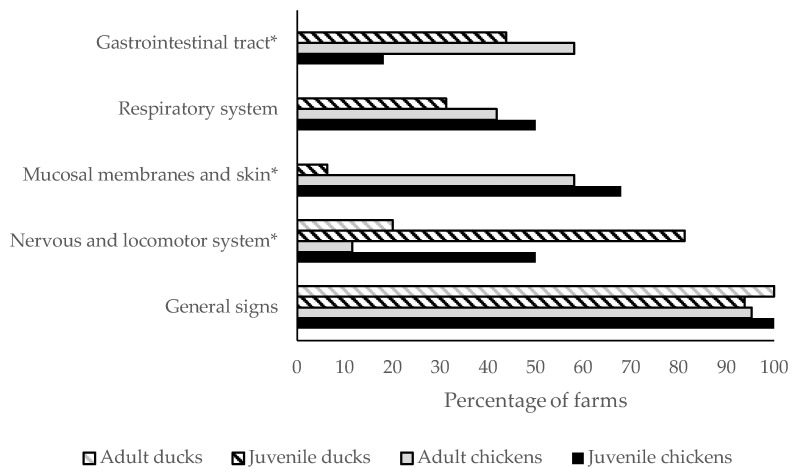
Overview of the frequency of detection of clinical signs, as categorized by organ system, observed on the day of clinical notification by the farm veterinarian, NVWA veterinarian, Royal GD veterinarian and/or farmer on HPAI-infected farms between 2014 and 2022. The clinical signs were scored per farm as observed or not observed. Juvenile chickens are included in layer-rearing and broiler farms; adult chickens are included in layer, layer breeder, broiler breeder and broiler grandparent farms; juvenile ducks are included in meat duck farms; and adult ducks are included in duck breeder farms. Absence of a bar means that the clinical signs were not observed in that category. Significant differences between different age groups according to Fisher’s exact tests (signs of the gastrointestinal tract (*p* < 0.001), mucosal membranes and skin (*p* < 0.001) and nervous and locomotor system (*p* < 0.001)) are indicated with *.

The frequencies of observed clinical signs are visualized in Figure 1 and Figures S5 and Table S3. No difference was found for signs of general disease and of the respiratory tract between adult chickens, juvenile chickens, adult ducks and juvenile ducks. Nervous and locomotor system signs were more often seen in juveniles than in adults in both ducks and chickens (*p* < 0.001). Signs of the mucosal membranes and skin were mainly seen in chickens, and more often in juveniles than in adults (*p* = 0.01). Gastrointestinal tract signs in chickens were more often seen in adults compared to juveniles, whereas in ducks the opposite trend was found (*p* < 0.001).

#### 3.2.3. Mortality

**Figure 2:**
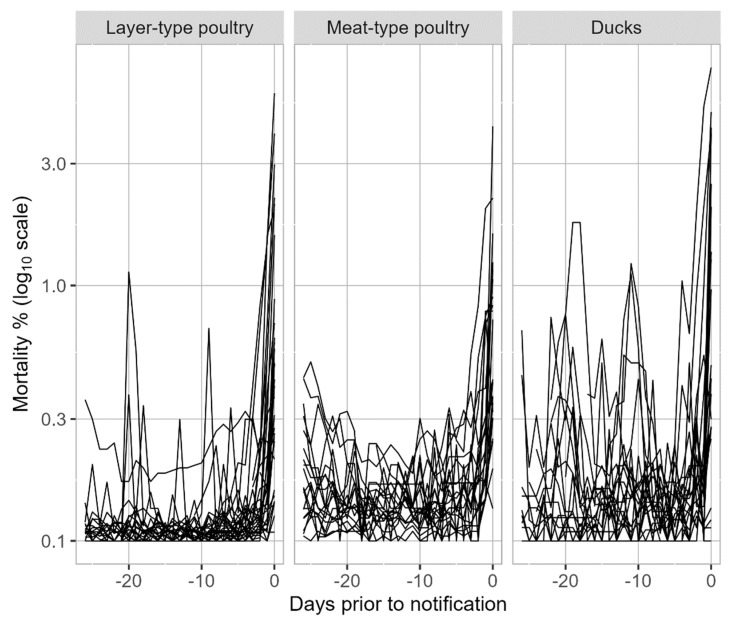
Mortality percentage expressed at a log10 scale from 26 days (−26) prior to the day of notification (day 0) in layer-type poultry (1 = layer-rearing, layer, layer breeder), meat-type poultry (2 = broiler, broiler breeder, broiler grandparent, turkey) and ducks (3 = meat duck and breeder duck) for each infected poultry house. The layer-type poultry group included 28 farms, of which 2 farms had two infected houses (*n* = 30). The meat-type poultry group included 15 farms, of which 3 farms had several infected houses (*n* = 21). The duck group consisted of 20 farms, of which 2 farms had two infected houses (*n* = 22). To avoid calculation problems, 0.1 was added to all percentages, with the lowest value of log10(0.1) = −1 meaning no mortality (0%).

An increase in mortality generally started within four days before notification (Figure 2). For both meat-type poultry and ducks, mean mortality was more often slightly higher within the 26 days before notification, whereas this was only seen for a few layer-type poultry farms. 

**Figure 3:**
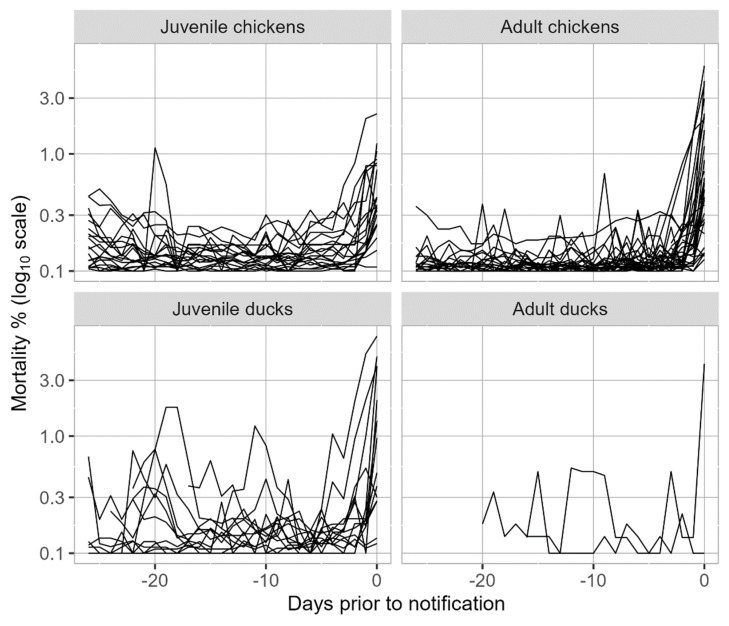
Mortality percentage expressed at a log10 scale from 26 days (−26) prior to the day of notification (day 0) for the different age groups: juvenile chickens (1 = layer-rearing and broiler), adult chickens (2 = layer, layer breeder, broiler breeder, broiler grandparent), juvenile ducks (3 = meat duck) and adult ducks (4 = duck breeder). The juvenile chicken group included 15 farms, of which 2 farms had several infected houses (*n* = 20). The adult chicken group included 29 farms, of which 2 farms had two infected houses (*n* = 31). The juvenile ducks included 12 farms of which 2 farms had two infected houses (*n* = 14). Adult ducks included of two farms (*n* = 2). To avoid calculation problems, 0.1 was added to all percentages, with the lowest value of log10(0.1) = −1 meaning no mortality (0%).

In juvenile chicken flocks, the mortality showed a more fluctuating trend within 26 days prior to notification compared to adult chicken flocks (Figure 3), which was also confirmed by differences in the mortality ratio (Table 5). The mortality in ducks was generally slightly higher and showed more peaks within 26 days before notification (Figure 3). With only two adult duck farms included in this dataset, mortality curves between adult and juvenile duck farms cannot be compared.

For 36 out of 92 farms, the MR could not be calculated due to limited data on mortality or the unavailability (unclear or unknown data) of reliable farm records. For farms with available mortality data, 83% of the layer-type chicken farms, 80% of the meat-type chicken farms and 54% of duck farms showed a MR > 3 the day before notification, but these differences were not statistically significant (Table 6 and Figure S6). 

**Figure 4:**
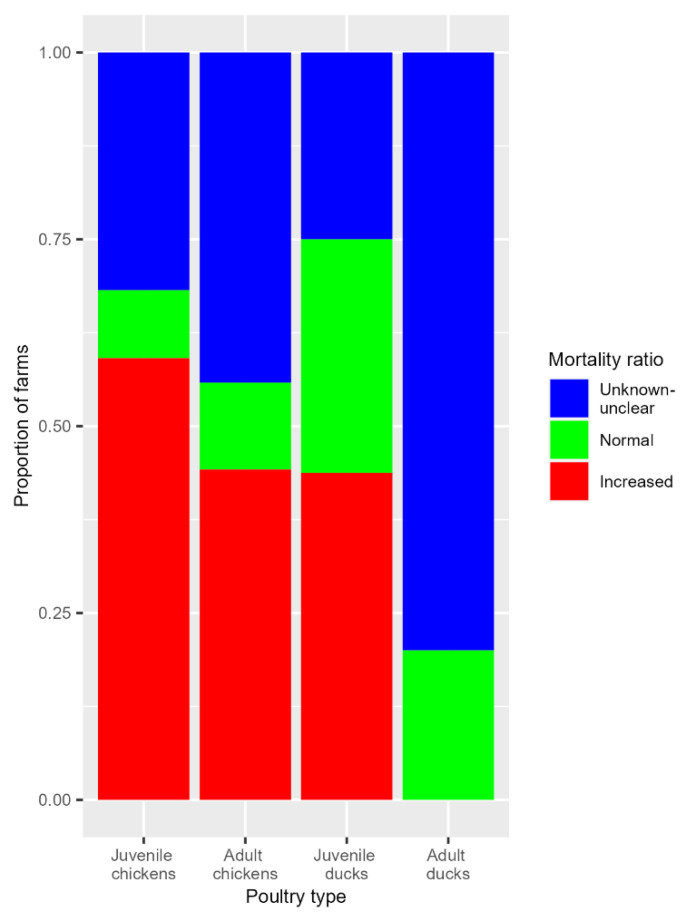
Stacked bars of the mortality ratios as the proportion of farms with juvenile chickens, adult chickens, juvenile ducks and adult ducks. Per farm, the mortality ratio one day prior to notification was calculated based on production charts and was scored as increased when the mortality ratio exceeded the cut-off value of 3, as not increased (MR < 3) or as unknown/unclear (when MR could not be calculated).

Most farms with juvenile chickens (87%), adult chickens (80%) and juvenile ducks (53%) had a MR > 3 the day before notification. An MR that did not exceed the cut-off value of 3 could only be calculated for one adult duck farm (Table 6 and Figure 4).

The percentage of layer farms infected with MR > 3 the day before notification was significantly higher for farms infected between 2014 and 2018 (88%) compared to layer farms in 2020–2022 (75%) (Table 6).

### 3.3. Clinical Signs during Outbreak Seasons 2014–2018 and 2020–2022

The clinical signs observed in layer farms, categorized per organ system and separated for 2014–2018 and 2020–2022, are summarized in Table 7, but none of the differences were statistically significant. Also, a visualization of the data of clinical signs per organ system for the different poultry types (layer- and meat-type poultry and ducks) (Figure S4) and age groups (adult chickens, juvenile chickens, adult ducks and juvenile ducks) (Figure S5) showed only limited differences in clinical manifestation between 2014 and 2018 and 2020 and 2022.

## 4. Discussion

This study describes the clinical manifestation of HPAI outbreaks with virus strains H5N1, H5N8 and H5N6 between 2014 and 2022 in different types of domestic poultry in the Netherlands. The affected poultry types were compared between outbreak seasons and differences in observed signs and mortality were studied for layer-type poultry, meat-type poultry and ducks, and between age groups. 

More meat-type poultry farms and fewer duck farms were HPAI-infected between 2020 and 2022 compared to 2014–2018. Adult ducks (breeders) and juvenile chickens (broilers and laying hen pullets) were not diagnosed with HPAI infection until 2020, while the percentage of infected adult chicken farms and juvenile duck farms decreased. Although an increase in outbreaks on meat-type poultry farms and a decrease on layer farms was a general trend in Europe between 2020 and 2022 [4,5,6,7,8], the relative increase in affected breeder duck farms and decrease in meat duck farms in the Netherlands was the opposite of the trend in Europe [4]. However, the seemingly changed distribution of HPAI-infected farms since 2020 can be explained by the structure of the poultry sector, as it was proportional to the different poultry types and age groups present in the Netherlands [33,34]. Genetic analyses indicated that the outbreaks since 2014 were mostly a result of separate single introductions from infected wild birds near to farms, with very limited to possibly no farm-to-farm transmission [29,30]. In contrast, most infected poultry farms between 2020 and 2022 in Germany were turkey meat farms [35,36], although turkey farms reflect a minority (1907 farms) in the German poultry sector compared to layers (47,104), broilers (3828) and ducks (4955) [37]. Phylodynamic and epidemiological outbreak analyses indicated that HPAI virus was likely introduced by infected wild birds near the German poultry farms, followed by farm-to-farm transmissions in dense farming areas, triggering major outbreaks and sustained continuous local circulation [38]. Massive farm-to-farm spread also occurred in the HPAI H7N7 epidemic in the Netherlands in 2003, where in the index case LPAI mutated to HPAI on a layer farm in a highly dense poultry area, resulting in 255 infected farms [39]. Because rare primary introductions can escalate towards a larger epidemic dominated by farm-to-farm transmission, especially in poultry-dense areas, high levels of biosecurity, early notification and the quick culling of infected birds remain of utmost importance.

With regard to differences in clinical signs between poultry types, we found that signs of the nervous and locomotor system were mainly reported in ducks, but also in meat-type poultry and less frequently on farms with layer-type poultry. Similar findings have been described in field and experimental studies for several different H5 HPAI viruses of clade 2.3.3.4., including H5N1 [40,41,42,43], H5N2 [44,45], H5N6 [46] and H5N8 [43,44,45,47,48]. Whereas we observed neurological and locomotor signs more often in broilers than in layers, in Nigeria, only layers, and not broilers showed these signs during the HPAI H5N1 and H5N8 outbreaks [43]. In the Dutch outbreaks, respiratory signs were more often reported in chickens, especially in meat-type poultry, than in ducks. 

Signs attributed to the reproductive tract were sometimes seen in layer-type poultry, and a decrease in egg production was reported in all five breeder duck flocks. In the study with only the 16 Dutch poultry farms infected with H5N8 or H5N6 between 2014 and 2018, signs of the reproductive tract were more often seen in chickens than in ducks, but this can be explained by the fact that only meat duck farms and no breeder duck farms were affected in that period [21]. Effects on reproduction were also found in duck flocks infected with HPAI H5N8 in Korea [48] and the UK in 2014 [49]. Elbers et al. (2021) also found a large and continuous drop in egg production in breeder duck flocks with H5 clade 2.3.4.4. HPAI viruses [50]. Signs of general disease, such as depression, hunched posture and ruffled feathers, were reported for all layer-type poultry, meat-type poultry and duck farms. Reduced feed and water intake was mainly seen in duck flocks, and was also seen in a HPAI H5N8-infected Pekin duck flock in California [47] and in the previously studied Dutch duck flocks [50]. No differences were found between layer-type poultry, meat-type poultry and ducks in the area of gastrointestinal signs. 

Age-group differences were also observed for the farms in this study. Nervous and locomotor system signs were more often seen in juveniles (both chickens and ducks) than in adults. Signs of the mucosal membranes and skin were mainly observed in chickens and rarely in ducks, and more often in juvenile chickens (broilers) compared to adult chickens (layers), which was also the case for the HPAI H5N8 outbreaks in the UK in 2020–2021 [13]. Adult chickens showed gastrointestinal signs more often than juveniles, whereas in ducks the opposite trend was found. General disease and respiratory tract signs were not significantly different between adult and juvenile chickens or ducks. However, the small number of adult duck farms (*n* = 5) does not allow for reliable comparisons to be made between juvenile and adult ducks. Moreover, as juvenile chickens included mostly broiler-type farms (17 broilers vs. 4 laying hen pullet farms), whereas the majority of adult chicken farms included layers (36 layer vs. 8 broiler breeder farms), the intertwining of genetic differences, differences in housing and management, and age, as well as the small sample size, limit conclusions that can be drawn from the comparisons. 

Several experimental studies have shown conflicting results with regard to the effect of age at time of infection. Clinical signs were subacute in 15-week-old male specific pathogen-free chickens infected with HPAI H5N2 but acute or peracute in older, 1-year-old, chickens [12]. Another study found significantly lower virus shedding in younger chickens (layers and broilers) than in older chickens after infection with HPAI H5N1 or LPAI H9N2 [51]. This was also seen in mallard ducks infected with LPAI H4N6 and H3N8 [52,53]. This may suggest increasing immunocompetence with age, resulting in a decreased susceptibility to pathological signs [51]. More severe neurological signs and 100% mortality were also found in 8-week-old Pekin ducks infected with H5N1, clade 2.2. virus, whereas 12-week-old Pekin ducks showed mild signs [54]. Also, the clinical signs and mortality coincided with an age-related association with viral loads and tissue distribution. The authors argue, however, that the high viral loads found in the liver, spleen and intestine in the older ducks, were not in line with a hypothesis of a more efficient immune system with aging and that more factors had to be in play [54]. Pantin-Jackwood et al. (2007) also found a higher mortality in 2-week-old ducks with more severe neurological signs compared to older 5-week-old ducks after inoculation with HPAI H5N1 [15]. In contrast, in a report of LPAI H4N6 in mallards it was suggested that older animals use more energy for reproduction at the cost of the immune response, making them more susceptible [53]. 

Generally, the increase in mortality on the farms started around four days before clinical suspicion was reported. The mortality ratio (MR) at the day before notification exceeded the cut-off value of 3 for most layer- and meat-type poultry farms, for both juvenile and adult chicken flocks. In duck flocks, especially for adults, an MR > 3 was observed less often. This is in line with reports of relatively limited mortality on duck farms with outbreaks of HPAI H5N1 or H5N8, including those from Egypt (2015) [44], Korea (2015) [48] and Nigeria (2021) [43]. The mean mortality was slightly higher and peaked more often in the 26 days before notification for ducks and meat-type broilers compared to layer-type farms. This can be explained by the fact that our dataset contained many juvenile (meat) duck farms and juvenile meat-type (broiler) poultry farms and mostly adult (laying-hen) layer-type flocks. A higher mean and greater fluctuating mortality in broilers and meat ducks is common in the first 10 days of life, and is related to hatching quality and management at placement on the farm [50,55]. This was also shown in the study of the 16 Dutch poultry farms between 2014 and 2018, where six out of seven meat duck farms reached the MR cut-off and more often showed an MR > 2.9 (the cut-off used in that study) within 30 days before notification compared to layers [21]. 

In our study, no differences in the observed clinical signs of different organ systems were found for the different outbreak seasons and virus strains between meat-type poultry, layer-type poultry and ducks and between different age groups. However, our analyses were hampered by the intertwinement of outbreak season, virus strains and affected poultry species and a limited number of farms with HPAI H5N8 and H5N6 compared to H5N1. Also, when we only compared laying hen flocks for 2014–2018 versus 2020–2022, no significant differences in clinical signs were found. Virus strain and species differences for viruses from the Dutch outbreaks were identified in other studies. For example, the 2014/15 H5N8, 2016/17 H5N8 and 2017/18 H5N6 viruses were all shown to be highly pathogenic for chickens, but both H5N8 viruses were mild in Pekin ducks compared to the 2017/18 H5N6 virus [56]. Another study with the Dutch 2017/18 HPAI H5N6 strain showed that systemic spread occurred in both ducks and chickens, but that virus distribution was more limited in ducks compared to chickens [46]. A study with the Dutch 2020/21 H5N8 virus indicated that the intravenous pathogenicity index (IVPI) was lower for Pekin ducks (1.74) compared to chickens (2.76) [29], although it should be noted that IVPI results may not always reflect clinical signs in practice [57]. Several studies with viruses from other countries revealed that the 2014/15 H5N8 clade 2.3.4.4.a virus caused fewer neurological signs and lower virus titers in the brain compared to the earlier 2.2. clade H5N1 viruses and the later clade 2.3.4.4.b viruses [58,59,60,61]. 

Differences between virus strains, host factors (genetics, age, immunity) and housing or environmental conditions under field or experimental circumstances make it difficult to compare studies and fully understand the pathobiology of HPAI infections. Moreover, many factors, such as the adaptation of the virus to the host, the immune response, tissue tropism, the infection dose and route, etc., result in differences in pathogenicity, infectivity and susceptibility. The interactions between all of these factors determine the transmission potential within and between flocks and at the wild bird–poultry interface [56,60,62]. The strong waterfowl adaptation of the 2016/17 HPAI H5N6 and 2021/22 HPAI H5N1 clade 2.3.4.4b viruses, for example, resulted in high infectivity and transmissibility in Anseriformes compared to Galliformes, and also compared to the 2014/15 H5N8 clade 2.3.4.4.a viruses [56,60,61]. Differences in manifestations between duck types have even been seen with viruses isolated at different timepoints from the same outbreak in the USA, i.e., the 2014/15 H5N2 clade 2.3.4.4 virus [45]. Hence, the monitoring of emerging viral genetic polymorphisms arising after transmission from Anseriformes to Galliformes are highly relevant for understanding the epidemiology and hence for mitigating measures and outbreak management [58,60].

Another important goal of this study was to highlight how HPAI can clinically manifest in poultry for different species, age groups, production types and virus types to improve awareness and facilitate the development of new protocols by policy makers and veterinarians that can assist farmers in recognizing infections and notifying agencies about them at an early stage. With regard to mortality, a small but nevertheless significantly lower percentage of infected layer flocks with a MR > 3 was found for 2020–2022 compared to 2014–2018. As the differences in IVPI for laying hens were limited over time, this may have been a result of earlier notifications by the farmer or veterinarian in 2020–2022 compared to 2014–2018. Potentially, this may be attributable to an increasing knowledge and awareness of clinical signs and mortality related to HPAI. Over the years, it has become apparent that, whereas in chicken flocks high mortality was the main reason for clinical notification, mortality remained limited in ducks and notifications were more often based on a decrease in feed or water intake or egg production. It was suggested that, in addition to a decrease in feed and water intake, a decrease in the egg production of breeder ducks >9% compared to the previous day was a more sensitive reporting threshold than mortality [50]. An analysis of mortality on eight broiler chicken flocks resulted in a proposed threshold for daily mortality of >0.17% (after the first week of production) for reporting, which was estimated to have reduced the delay in reporting with 5 days. This was higher than the threshold suggested for layers (0.13%) and lower than that suggested for ducks (0.3%), reflecting the differences in levels of mortality between poultry types [55]. The work on mortality thresholds for laying hens [21,25] and these studies prompted Dutch policy makers to adapt the notification thresholds. Instead of only having to report after reaching 3% mortality in one week or 0.5% per day for two consecutive days, more sensitive thresholds were added (see Appendix A), with obligations to report at MR > 3 in chickens and for ducks 0.15% per day for two days or 0.5% per day combined with a feed intake reduction of 5%. However, although such policy changes are a great step, it is primarily essential that farmers keep detailed farm records. For meat duck and breeder farms, limited data were available on feed and water intake, and on egg production for breeders. In addition to adapting reporting thresholds, relevant action from a policy perspective may include obligations on farm record keeping. 

## 5. Conclusions

The outcomes of this study can provide starting points for adjusting the early warning criteria currently used for the early detection and notification of signs of HPAI infection in poultry flocks. Whereas in layers and breeder chicken flocks the most dominant early sign of infection is exponential increasing mortality, mortality can be limited in adult flocks, or show a more fluctuating trend in juvenile ducks and chickens. In ducks, drops in feed or water intake or egg production may be the only initial signs of infection, indicating that the criteria for reporting should be different for different poultry species and age groups. Until suitable vaccines and vaccination programs are in place that can successfully reduce the risk of infection and transmission between flocks, most European countries will rely on stamping-out policies. Under these circumstances, not only early reporting, but also quick culling and strict hygiene protocols are essential to reduce the risk of further spread to other farms, especially when farms share many contacts or are located in a poultry-dense area and farm-to-farm spread is likely to occur. 

## Figures and Tables

**Table 1:** Distribution of HPAI-infected poultry farms for each outbreak season between 2014 and 2022.

		Layer Rearing	Layer	Broiler	Broiler Breeder	Broiler Grandparent	Turkey	Meat Duck	Duck Breeder	Total
**Poultry type**		Layer-type ^1^		Meat-type ^2^				Ducks ^3^		
**Age group**		Ch-Juv ^4^	Ch-Ad ^5^	Ch-Juv ^4^	Ch-Ad ^5^	Ch-Ad ^5^		D-Juv ^6^	D-Ad ^7^	
**Outbreak**	**Virus**	*n*	*n*	*n*	*n*	*n*	*n*	*n*	*n*	*n*
2014–2015	H5N8	0	3	0	1	0	0	1	0	5
2016–2017	H5N8	0	4	0	0	0	0	4	0	8
2017–2018	H5N6	0	1	0	0	0	0	2	0	3
2020–2021	H5N8	0	4	2	1	0	2	1	0	10
2021–2022	H5N1	4	24	15	5	1	4	8	5	66
Total (n)		4	36	17	7	1	6	16	5	92

^1^ Layer rearing, layer and layer breeder; ^2^ broiler, broiler breeder, broiler grandparent and turkey; ^3^ meat duck and duck breeder; ^4^ Ch-Juv = juvenile chickens (layer rearing and broiler); ^5^ Ch-Ad = adult chickens (layer, broiler breeder, broiler grandparent); ^6^ D-Juv = juvenile duck (meat duck); ^7^ D-Ad = adult duck (duck breeder); n = number of farms.

**Table 2:** The availability of data sources.

Data Source	Number of Farms ^1^	Total ^2^
Visit reports from the Royal GD	61	92
Visit reports the NVWA	83	92
Production charts	63	92
Detailed epidemiological investigation	36	92
Online survey	16	92

^1^ Number of farms with available data sources; ^2^ total number of farms.

**Table 3:** Categorization of poultry farms for the analyses and the number of farms per subgroup.

Poultry	Farms (*n*)	Order	Genus	Poultry Type	Age ^1^
Layer rearing	4	Galliformes	*Gallus gallus*	1: Layer (*n* = 40)	1A: Chicken Juv (*n* = 4)
Layer	35				1B: Chicken Ad (*n* = 36)
Layer breeder ^2^	1				
Broiler	17			2: Meat (*n* = 31)	2A: Chicken Juv (*n* = 17)
Broiler breeder	7				2B: Chicken Ad (*n* = 8)
Broiler grand- parent	1				
Turkey	6		*Meleagris gallopavo domesticus*		
Meat duck	16	Anseriformes	*Anas platyrhynchos domestica*	3: Ducks (*n* = 21)	3A: Duck Juv (*n* = 16)
Duck breeder	5				3B: Duck Ad (*n* = 5)
Total (*n*)	92				

^1^ Ad = adult; Juv = juvenile; *n* = number of farms. ^2^ The farm was a facility for eggs for vaccine production, which are comparable, with regard to management, to layer-breeder farms.

**Table 4:** Distribution of HPAI-infected poultry farms categorized according to different poultry types and age groups (only chickens and ducks) for all outbreaks between 2014 and 2018 and 2020 and 2022 combined.

Season	2014–2018		2020–2022	
Poultry type *	*n*	%	*n*	%
Layer-type ^1^	8	50.0	32	42.1
Meat-type ^2^	1	6.3	30	39.5
Ducks ^3^	7	43.8	14	18.4
Total poultry type (n)	16	100.0	76	100.0
Age group *	*n*	%	*n*	%
Chicken juvenile ^4^	0	0.0	22	31.4
Chicken adult ^5^	9	56.2	34	48.7
Duck juvenile ^6^	7	43.8	9	13.0
Duck adult ^7^	0	0.0	5	7.1
Total age group (*n*)	16	100.0	70	100.0

^1^ Layer-rearing, layer, layer breeder; ^2^ broiler, broiler breeder, broiler grandparent, turkey; ^3^ meat duck and breeder duck, ^4^ layer-rearing, broiler; ^5^ layer, layer breeder, broiler breeder, broiler grandparent; ^6^ meat duck; ^7^ duck breeder’; *n* = number of farms. Overall differences in distribution of the different poultry types (layer-type, meat-type and ducks) and age groups between 2020 and 2022 and 2014 and 2018 were both significant (Fisher’s exact tests *p* = 0.01 and *p* = 0.00, respectively) and therefore indicated with *.

**Table 5:** Number (%) of HPAI-infected farms with observed clinical signs between 2014 and 2022 categorized for different poultry types and per organ system.

Clinical Signs ^4^	Layer-Type ^1^			Meat-Type ^2^			Ducks ^3^			Total
	*n*	*n* (Total)	%	N	*n* (Total)	%	*n*	*n* (Total)	%	n
General signs	38	40	95.0	31	31	100.0	20	21	95.2	89
Respiratory system *	15	40	37.5	19	31	61.3	5	21	23.8	39
Nervous and locomotor system *	6	40	15.0	15	31	48.4	14	21	66.7	35
Mucosal membranes and skin *	22	40	55.0	21	31	67.8	1	21	4.7	44
Gastrointestinal tract	22	40	55.0	13	31	41.9	7	21	33.3	42
Reproductive tract *	7	40	19.4	2	7	28.6	5	5	100.0	14
Total (n)	110			101			52			

^1^ Layer-rearing, layers, layer breeder; ^2^ broilers, broiler breeders, broiler grandparent, turkeys; ^3^ meat ducks and breeder ducks; ^4^ clinical signs observed by veterinarians of the NVWA and/or the Royal GD and/or the farmer scored as observed vs. not observed; *n* = the number of farms with the observed clinical signs; *n* (Total) = the total number of farms with layer-type poultry, meat-type poultry or ducks; % = the percentage of farms. Signs for which significant differences in occurrence were found between poultry types according to Fisher’s exact tests (respiratory signs (*p* = 0.02), nervous and locomotor system (*p* = 0.00), mucosal membranes and skin (*p* = 0.00) and reproductive tract (*p* = 0.00)), are indicated with *.

**Table 6:** The number (%) of farms exceeding MR > 3 in different poultry types and age groups and layers for the seasons 2014–2018 and 2020–2022.

Mortality Ratio > 3	n (MR > 3) ^1^	*n* (Total) ^1^	%
**Poultry type**			
Layer-type	19	23	82.6
Meat-type	16	20	80.0
Ducks	7	13	53.8
**Age groups**			
Juvenile chickens	13	15	86.7
Adult chickens	19	24	79.2
Juvenile ducks	7	12	58.3
Adult ducks	0	1	0.0
Layers *			
2014–2018	7	8	87.5
2020–2022	9	12	75.0

^1^ n (MR > 3) = the number of farms with mortality ratio (MR) > 3; n (Total) = the number of farms with available mortality ratio data; % = the percentage of farms with MR > 3. A significant difference in the percentage of farms with MR > 3 according to the Fisher’s exact test, indicated with *, was only found for layer farms diagnosed between 2014 and 2018 compared to 2020 and 2022 (*p* = 0.01), but not between poultry types or age groups.

**Table 7:** Frequency (%) of farms observed with clinical signs on HPAI-infected layer farms in 2014–2018 and 2020–2022 per organ system.

Clinical Signs ^1^	2014–2018			2020–2022		
	*n*	*n* (Total)	%	*n*	*n* (Total)	%
General	7	8	87.5	27	28	96.4
Respiratory	3	8	37.5	11	28	39.3
Nervous and locomotor system	1	8	12.5	2	28	7.1
Mucosal membranes and skin	4	8	50.0	15	28	53.4
Gastrointestinal tract	5	8	62.5	17	28	60.7
Reproductive tract	1	8	12.5	6	28	21.4
Mucosal membranes and skin	4	8	50.0	15	28	53.4
Total (*n*)	25			93		

^1^ Clinical signs observed by veterinarians of the NVWA and/or the Royal GD and/or the farmer scored as observed vs. not observed; *n* = the number of farms with observed clinical signs, n (Total) = the total number of layer farms; % = the percentage of farms.

## Data Availability

Some of the recent data with regard to infected farms in this study are available from the following resources available in the public domain [Wageningen Bioveterinary Research Bird Flu Updates. for 2020/2021: https://www.wur.nl/en/research-results/research-institutes/bioveterinary-research/show-bvr/bird-flu-at-poultry-farms.htm, accessed on 4 March 2024 and for 2021–2022: Wageningen Bioveterinary Research Bird Flu Updates: https://www.wur.nl/en/research-results/research-institutes/bioveterinary-research/show-bvr/bird-flu-at-poultry-farms-in-20212022.htm, accessed on 4 March 2024], or have been published in other published reports referred to in this manuscript. The other types of data, including details on findings during official visits and diagnostic test results by the competent authority, contain information that cannot be made public. These can be linked to the previously mentioned publicly available resources, and its publication would therefore be in violation of EU and national regulations and confidentiality agreements between the researchers and the competent authority. Requests for access to specific parts of the datasets, provided this request can be granted within the aforementioned restrictions, should be directed to the corresponding authors.

## Supplementary Materials

The following supporting information can be downloaded at: https://www.mdpi.com/article/10.3390/pathogens13040280/s1, Figure S1: Distribution of HPAI-infected farms for each outbreak season between 2014 and 2022; Figure S2: Number of HPAI-infected farms between 2014 and 2022 with observed clinical signs categorized for different poultry types and per organ system; Figure S3: Overview of the frequency of detection of clinical signs, as categorized by organ system, observed on the day of clinical notification by the farm veterinarian, NVWA veterinarian, GD veterinarian and/or farmer on HPAI-infected farms between 2014 and 2022; Figure S4: Overview of the frequency of detection of clinical signs, as categorized by organ system, in layer-type poultry, meat-type poultry and ducks observed on the day of clinical notification by the farm veterinarian, NVWA veterinarian, GD veterinarian and/or farmer on HPAI-infected farms between 2014 and 2022; Figure S5: Overview of the frequency of detection of clinical signs, as categorized by organ system, in juvenile and adult chickens and ducks observed on the day of clinical notification by the farm veterinarian, NVWA veterinarian, GD veterinarian and/or farmer on HPAI-infected farms between 2014 and 2022; Figure S6: Stacked bars of the mortality ratio’s as proportion of farms in layer-type poultry, meat-type poultry and ducks; Table S1: List of clinical signs scored by veterinarians of the NVWA; Table S2: List of clinical signs categorized per organ system; Table S3: Number (%) of farms infected between 2014 and 2022 with observed clinical signs categorized by different age groups and per organ system.

## Appendix A

The “Decree animal keepers” [Besluit houders van dieren: https://wetten.overheid.nl/BWBR0035217/2024-01-01, accessed on 4 March 2024] enforces Regulation (EU) 2016/429 on transmissible animal diseases, and states that suspicions of avian influenza need to be notified to authorities by farmers, veterinarians or laboratory workers. The Dutch Statutory Regulation “Regulation animal keepers” [Regeling houders van dieren: https://wetten.overheid.nl/BWBR0035248/2023-11-16, accessed on 4 March 2024] states that farmers need to consult their veterinarians in case AI-susceptible animals show clinical signs or when specific reporting thresholds are met, i.e., when for two consecutive days the feed or water intake decreases by 5% or more, or when the egg production in laying hens and breeders decreases by 5% per day or more for two consecutive days. In addition, farmers are required to report increased mortality in laying hens, broilers and broiler breeders older than 10 days to the authorities at >0.5% per flock for two consecutive days or if mortality is >3% in a single week. Since the 20th of November 2020, additional rules also apply. These are part of the “Regulation on veterinary measures for specific animal diseases or zoonoses” [Regeling veterinaire maatregelen specifieke dierziekten of zoönosen: https://wetten.overheid.nl/BWBR0045051/2024-01-29, accessed on 4 March 2024], which is based on the Animal Act and Regulation (EU) 2016/429 on transmissible animal diseases. This regulation stipulates that duck farmers are required to report (for flocks older than seven days) a daily mortality of >0.15% for two consecutive days or when daily mortality is >0.5% for one day in combination with a decrease in feed intake of 5%. Broiler, laying hen and breeder farmers are required to report increased mortality when the mortality rate is tripled for two consecutive days compared to the average mortality in the week before [21,25].
