# Supplementary material for: Comparison of the Clinical Manifestation of HPAI H5Nx in Different Poultry Types in the Netherlands, 2014–2022
Source: Pathogens. 2024 Mar 26;13(4):280. doi: 10.3390/pathogens13040280 (PMC11055007; doi:10.3390/pathogens13040280)
Supplement: Supplementary file 1 [file pathogens-13-00280-s001.zip › pathogens-2868314-supplementary.pdf]

Supporting information

Comparison of the clinical manifestation of HPAI H5Nx in different poultry types in the Netherlands, 2014-2022

Wendy J. Wolters, J.C.M. Vernooij, Thomas Spliethof, Jeanine Wiegel, Armin R.W. Elbers, Marcel A.H. Spierenburg, J. Arjan Stegeman and Francisca C. Velkers

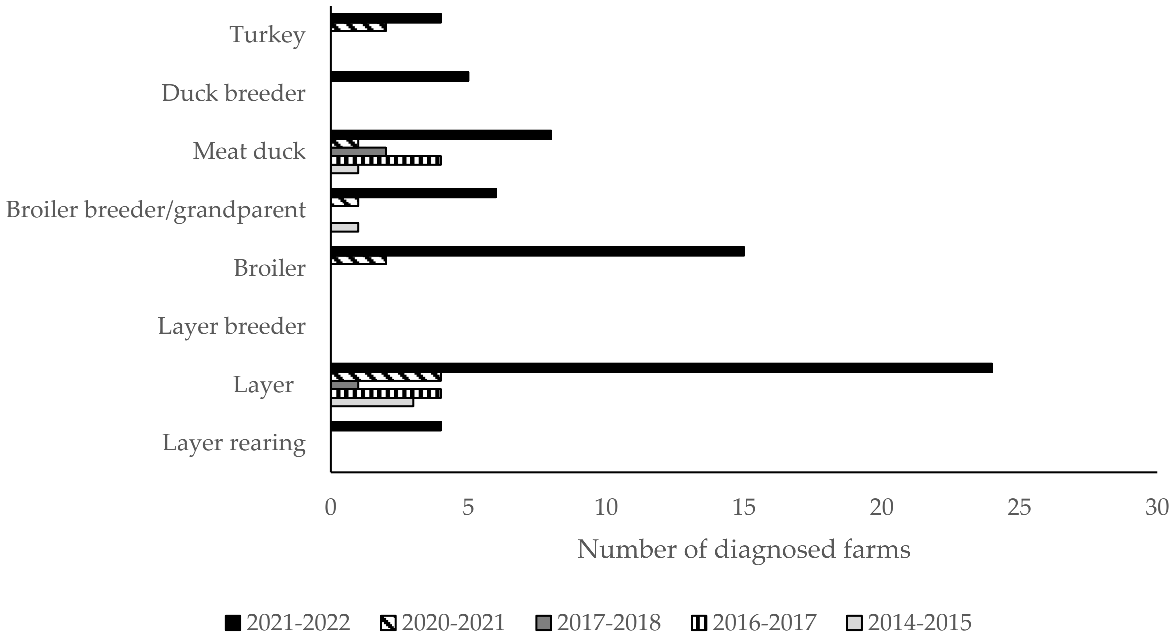

Figure S1. Distribution of HPAI infected farms for each outbreak season between 2014-2022.

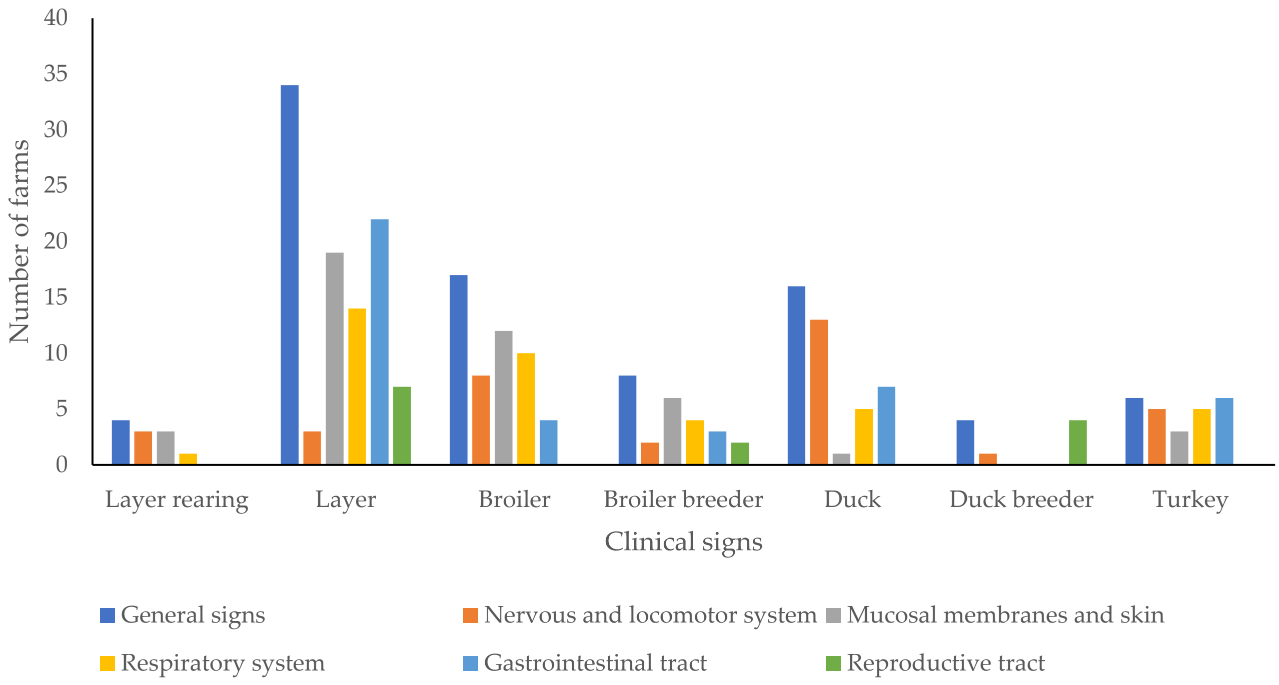

Figure S2. Number of HPAI infected farms between 2014-2022 with observed clinical signs categorized for different poultry types and per organ system. Clinical signs were observed by veterinarians of the NVWA and/or the Royal GD and/or the farmer scored as observed vs not observed before or right after notification.

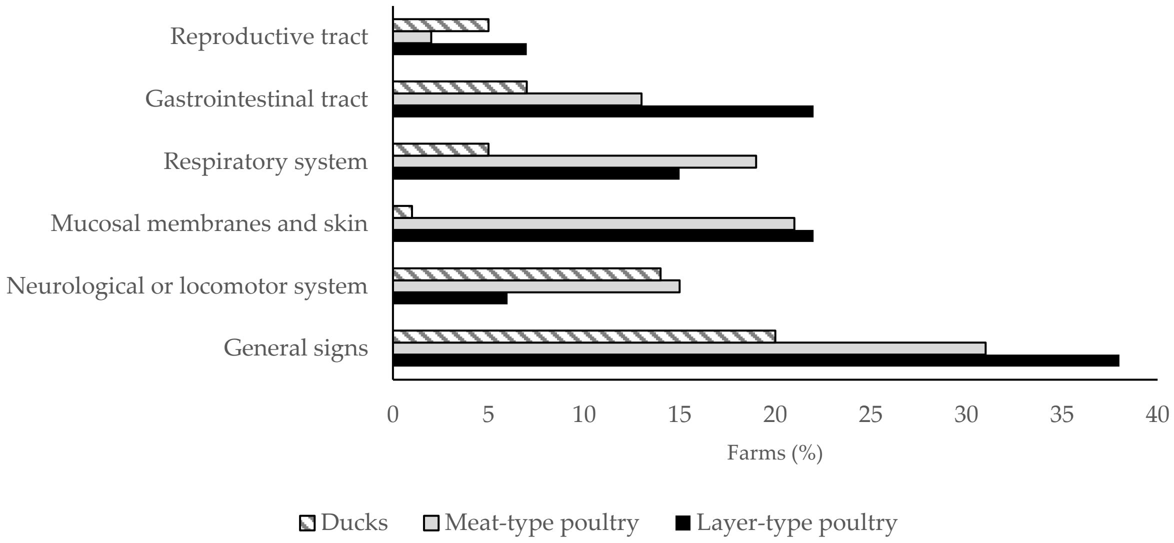

Figure S3. Overview of the frequency of detection of clinical signs, as categorized by organ system, observed on the day of clinical notification by the farm veterinarian, NVWA veterinarian, GD veterinarian and/or farmer on HPAI infected farms between 2014-2022. The clinical signs were scored per farm as observed or not observed. Only significant differences were found in the occurrence of signs of the mucosal membranes and skin (Fisher's exact test, $p = 0.00$), signs of the nervous and locomotor system (Fisher's exact test, $p = 0.00$), respiratory signs (Fisher's exact test, $p = 0.02$) and signs of the reproductive tract (Fisher's exact test, $p = 0.00$) between the different poultry types.

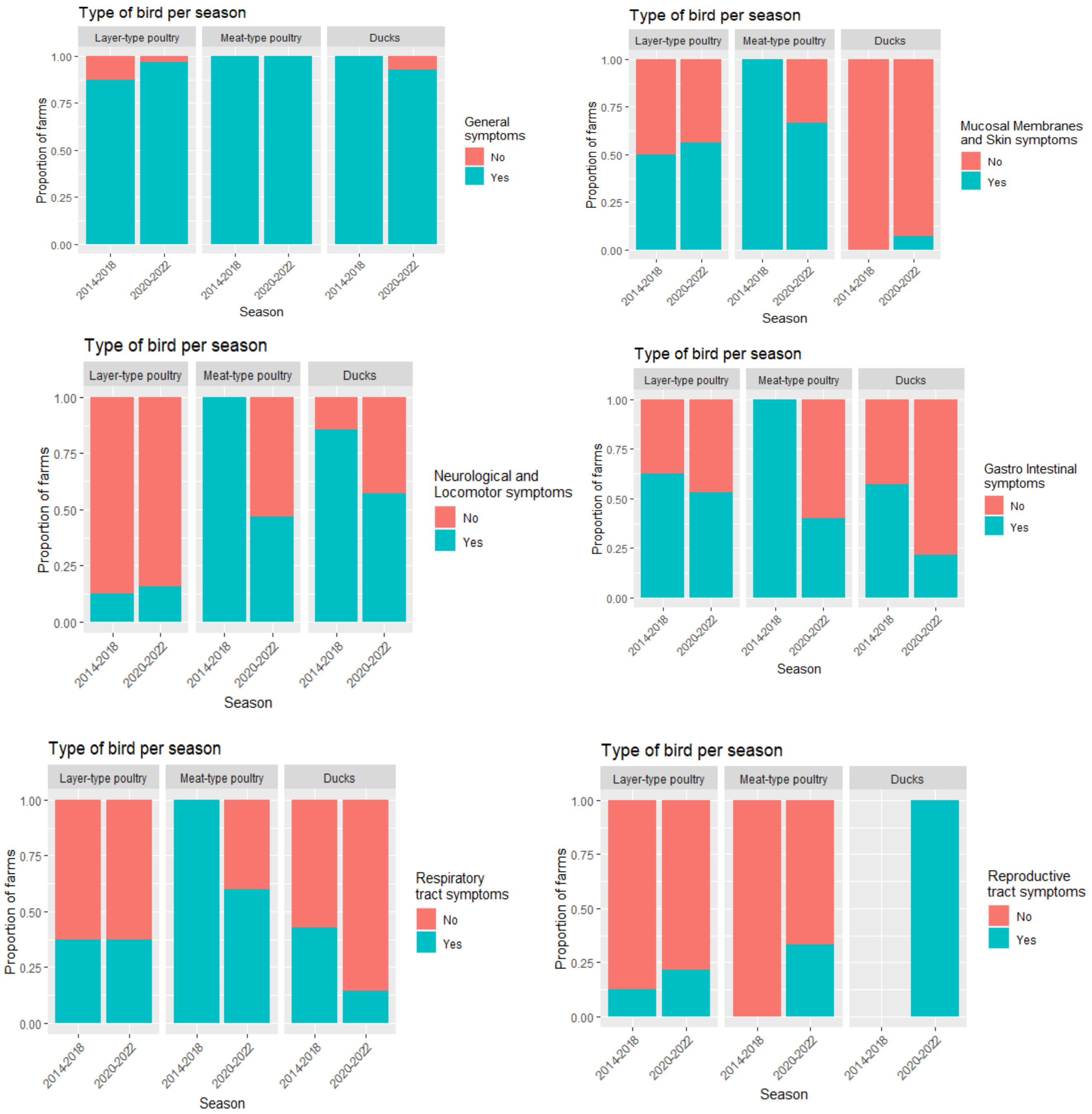

Figure S4. Overview of the frequency of detection of clinical signs, as categorized by organ system, in layer-type poultry, meat-type poultry and ducks observed on the day of clinical notification by the farm veterinarian, NVWA veterinarian, GD veterinarian and/or farmer on HPAI infected farms between 2014-2022. The clinical signs were scored per farm as observed (Yes) or not observed (No).

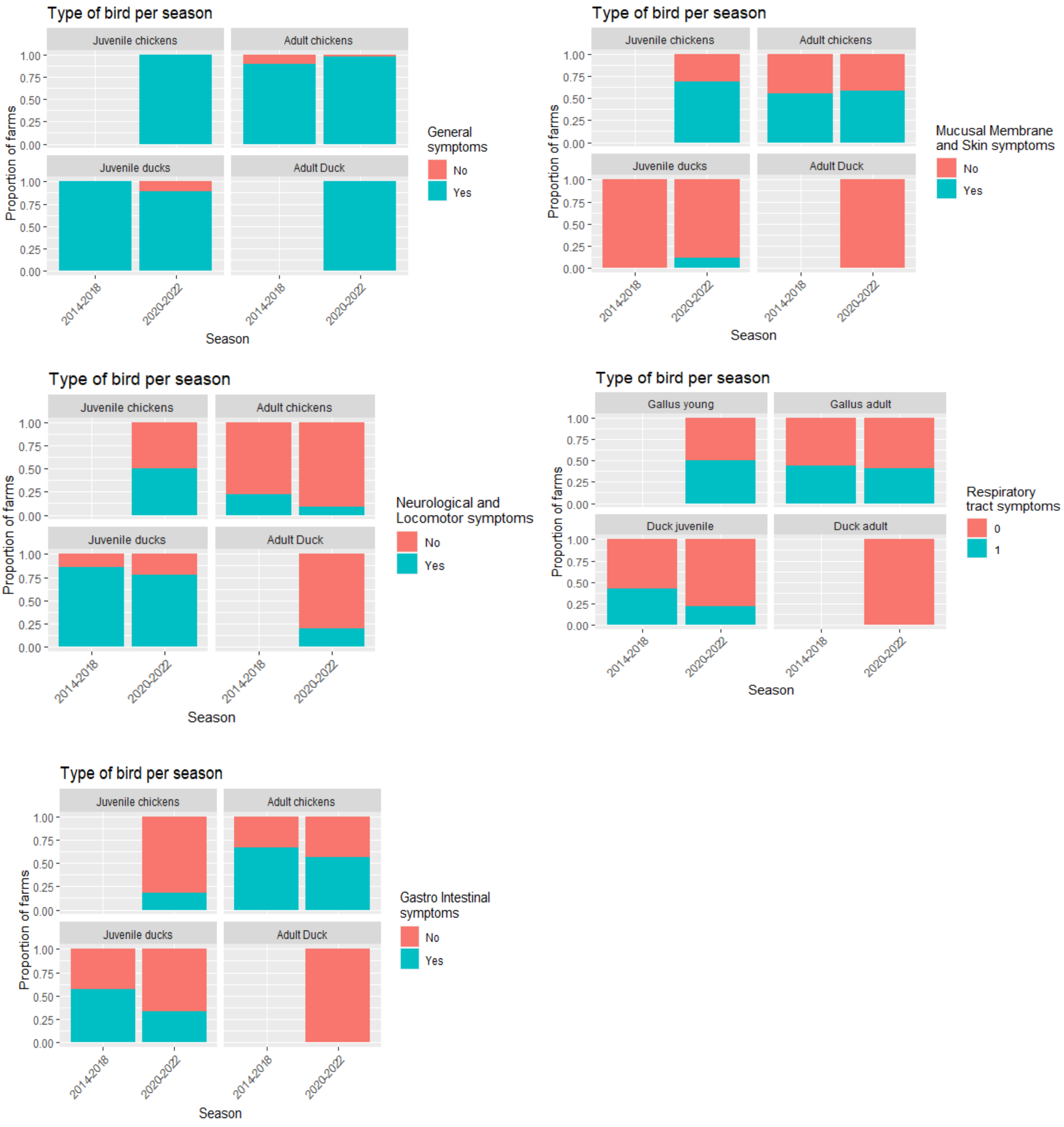

Figure S5. Overview of the frequency of detection of clinical signs, as categorized by organ system, in juvenile and adult chickens and ducks observed on the day of clinical notification by the farm veterinarian, NVWA veterinarian, GD veterinarian and/or farmer on HPAI infected farms between 2014-2022. The clinical signs were scored per farm as observed (Yes) or not observed (No). Signs of the reproductive tract were not included as juvenile chickens and juvenile ducks are not egg producing flocks.

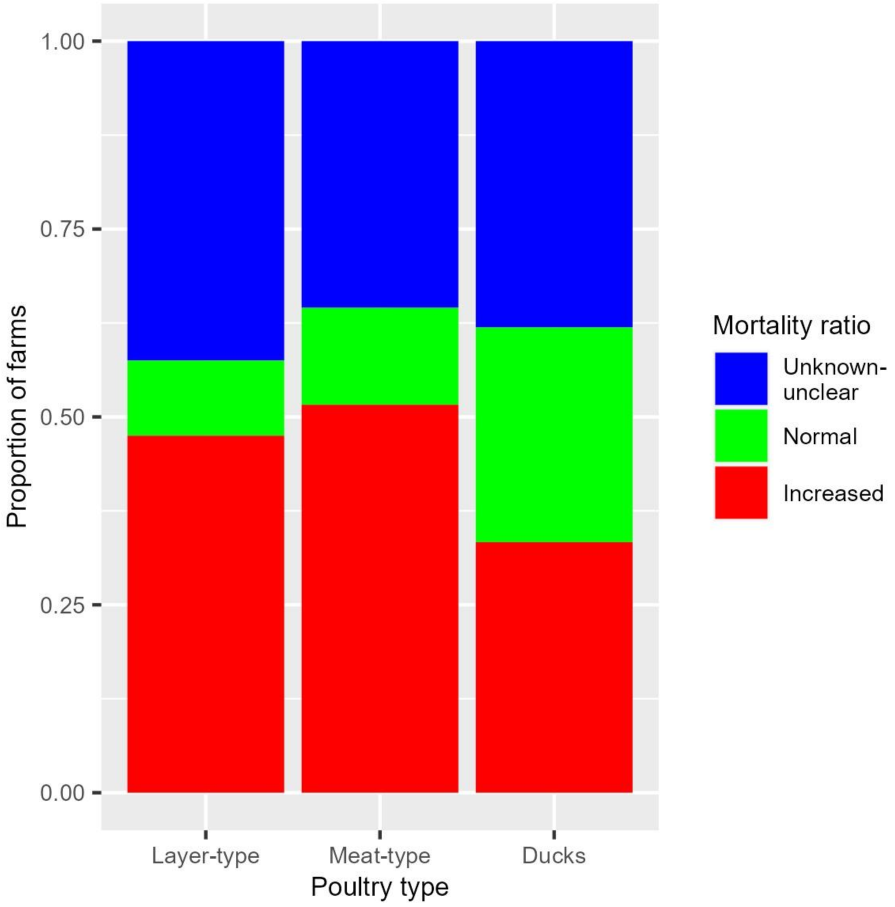

Figure S6. Stacked bars of the mortality ratio's as proportion of farms in layer-type poultry, meat-type poultry and ducks. Per farm the mortality ratio one day prior to notification was calculated based on production calendars and was scored whether the mortality ratio exceeded the cut off value of 3, as not increased (MR<3) or as unknown/unclear (when MR could not be calculated).

**Table S1.** List of clinical signs scored by veterinarians of the NVWA
Anorexia
Sudden death / increased water intake
Ruffled feathers / hunched posture
Diarrhea
Reduced feed intake / decrease in egg production / mottled or pale eggs
Edema of the neck, head and eyes
Swollen wattles, combs and extremities
Cyanosis of the wattles, combs and extremities
Excessive lacrimation
Hemorrhagic conjunctiva
Neurological signs
Respiratory signs / inactivity

Table S2. List of clinical signs categorized per organ system

General signs	Respiratory system	Nervous and locomotor system	Mucosal membranes and skin	Gastrointestinal tract	Reproductive tract
Decreased activity / depression	Rales/rattles	Tremors	Cyanosis of the wattles	Diarrhea	Decreased egg production
Reduced vocalization	Sneezing	Paralysis	Hemorrhagic conjunctiva		Decreased egg-shell quality
Warm extremities	Excessive lacrimation	Torticollis	Swollen head		Pale or mottled eggs
Cold extremities	Nasal discharge	Opisthotonus	Swollen wattles		
Shivering	Sinusitis	Abnormal gait	Dark small combs		
Reduced feed intake	Conjunctivitis	Lameness	Cyanosis extremities		
Reduced water intake	Dyspnea	Ataxia			
Increased water intake		Inability to stand			
Decreased daily growth					
Hunched posture					
Ruffled feathers					
Closed eyes					
Sudden death					

Signs caused by endothelial damage were attributed to the mucosal membranes and skin such as edema of neck, eyes and head including wattles and combs, hemorrhages and cyanosis of wattles, combs and extremities. Respiratory signs included for instance dyspnea, conjunctivitis, excessive lacrimation, nasal discharge and rales/rattles. Signs of the locomotor system and nervous system were combined and included for instance tremors, paralysis, lameness, torticollis and opisthotonos. For the egg-producing farms, including breeder farms and layer farms, it was recorded whether the egg production was decreased including the presence of pale or mottled eggs and reduced egg-shell quality.

Table S3. Number (%) of farms infected between 2014-2022 with observed clinical categorized for different age groups and per organ system

Clinical signs[5]	Juvenile chickens[1]		Adult chickens[2]		Juvenile ducks[3]		Adult ducks[4]	
	n	%	n	%	n	%	n	%
General	22	100.0	41	95.3	15	93.8	5	100.0
Mucosal membranes and skin	15	68.0	25	58.1	1	6.3	0	0.0
Nervous and locomotor system	11	50.0	5	11.6	13	81.3	1	20.0
Respiratory system	11	50.0	18	41.9	5	31.3	0	0.0
Gastrointestinal tract	4	18.2	25	58.1	7	43.8	0	0.0

[1]Layer rearing, broiler; [2] Layers, layer breeder, broiler breeder, broiler grandparent; [3] Meat duck; [4] Duck breeder; [5] Clinical signs observed by veterinarians of the NVWA and/or the Royal GD and/or the farmer scored as observed vs not observed before or right after notification; [5] Includes only the egg producing farms (layers, layer breeder, broiler breeder, broiler grandparent and breeder duck; n = the number of farms, % = the percentage of farms. Only significant differences were found in the occurrence of signs of the mucosal membranes and skin (Fisher's exact test, p = 0.00), signs of the nervous and locomotor system (Fisher's exact test, p = 0.00), and signs of the gastrointestinal tract (Fisher's exact test, p = 0.00) between the different age groups.
